# Quantum Mechanics Characterization of Non-Covalent Interaction in Nucleotide Fragments

**DOI:** 10.3390/molecules29143258

**Published:** 2024-07-10

**Authors:** Mayar Tarek Ibrahim, Elizabeth Wait, Pengyu Ren

**Affiliations:** 1 Department of Biomedical Engineering, The University of Texas at Austin, Austin, TX 78712, USA; mayar.mohamed@austin.utexas.edu; 2 Interdisciplinary Life Sciences Graduate Program, The University of Texas at Austin, Austin, TX 78712, USA; elizabethewait@austin.utexas.edu

**Keywords:** nucleotides, ALMO EDA2, SAPT2+(3)/dmp2, dispersion, induction

## Abstract

Accurate calculation of non-covalent interaction energies in nucleotides is crucial for understanding the driving forces governing nucleic acid structure and function, as well as developing advanced molecular mechanics forcefields or machine learning potentials tailored to nucleic acids. Here, we dissect the nucleotides’ structure into three main constituents: nucleobases (A, G, C, T, and U), sugar moieties (ribose and deoxyribose), and phosphate group. The interactions among these fragments and between fragments and water were analyzed. Different quantum mechanical methods were compared for their accuracy in capturing the interaction energy. The non-covalent interaction energy was decomposed into electrostatics, exchange-repulsion, dispersion, and induction using two ab initio methods: Symmetry-Adapted Perturbation Theory (SAPT) and Absolutely Localized Molecular Orbitals (ALMO). These calculations provide a benchmark for different QM methods, in addition to providing a valuable understanding of the roles of various intermolecular forces in hydrogen bonding and aromatic stacking. With SAPT, a higher theory level and/or larger basis set did not necessarily give more accuracy. It is hard to know which combination would be best for a given system. In contrast, ALMO EDA2 did not show dependence on theory level or basis set; additionally, it is faster.

## 1. Introduction

Nucleotides are the main building blocks of nucleic acids, which include DNA and RNA. Nucleotides are composed of a neutral sugar moiety (deoxyribose in DNA or ribose in RNA), a negatively charged phosphate group, and nucleobase. Nucleobases are either purine (Adenine (A), Guanine (G)), or pyrimidine (Cytosine (C), Thymine (T), or Uracil (U)) [1]. Nucleic acids are not only essential for the storage of genetic material, transcription, and translation but also for various cellular processes, such as ligand binding, enzymatic function, regulation of metabolic activities, genomic instability, apoptosis, and stress response [2,3,4,5].

The most common configuration of DNA is the B-DNA duplex [6,7]. The transition from B-DNA geometry to A-DNA geometry is observed upon the addition of solvents or interaction with other proteins or drug molecules [8,9,10]. The Z-DNA form has been detected in crystal structures and environments with high salt concentrations [11,12,13]. RNA is predominantly found in A-form helices, as well as hairpin loops and tertiary structures, depending on the sequence and the number of strands [14]. Depending on whether the nucleotide is in the B-DNA or A-DNA geometry, the sugar moiety adopts either C2′-endo conformation or C3′-endo conformation, respectively [15]. These varied and complex configurations of nucleic acids are maintained and stabilized by the non-covalent interactions among the three main components of the nucleotides. The two main types of non-covalent interactions present in nucleic acids are hydrogen bonds and π-stacking interactions.

Due to the complex nature of DNA and RNA molecules and the dominance of non-covalent interactions, molecular simulations using the current molecular mechanics (MM) forcefields have been challenging. Current nucleic acid forcefields treat the non-covalent interactions as the sum of electrostatic interactions between fixed atomic charges and van-der-Waals interactions (accounted for by Lennard-Jones potentials) [16]. Theoretically, the fixed charges are parameterized to capture the electrostatics of biomolecules in a solvated environment. Therefore, it is not expected that such force fields can be validated on small molecular clusters in the gas phase. On the other hand, the AMOEBA (atomic multipole optimized energetics for biomolecular applications) forcefield employs polarizable atomic multipoles to improve the representation of electrostatics [11,17,18]. Drude NA force fields account for polarization in NAs based on classical Drude oscillators [19,20,21]. While these more sophisticated descriptions of many-body polarization significantly improved the accuracy of classical mechanics models of biomolecules, challenges remain.

For example, in the case of stacked aromatic molecules, neither point charge nor multipole models are able to capture electrostatic interactions between the atomic rings [22]. This is attributed to the lack of charge penetration effect needed to account for the π-stacking interactions due to the overlapping of the π-electron clouds and atomic nuclei [23,24,25]. Such effect can be clearly seen from the ab-initio energy decomposition methods such as Symmetry-Adapted Perturbation Theory (SAPT) [26,27] and Absolutely Localized Molecular Orbitals (ALMO) [28,29,30]. The improvement in the accuracy of molecular mechanics models depends on the quality of the underlying ab initio quantum mechanics (QM) benchmarks, which are used for designing the physical function forms and deriving force field parameters. The QM benchmarks are also commonly used for training machine learning potentials [31,32,33,34,35,36,37,38].

For molecular interaction and conformation energy, the CCSD(T) method with complete basis set (CBS) is considered the gold standard for QM, with excellent agreement to the experimental results for atomic properties and is commonly used for evaluating the accuracy of other QM methods such as MP2 and DFTs [39,40,41,42,43]. There are several databases that report molecular interaction energy from CBS-CCSD(T) and other QM methods [38,44,45]. One such database consisting of thousands of dimer interactions published by Donchev et al. at DEShaw used CCSD(T) as the benchmark whenever possible [44]. When computational expense did not allow for CCSD(T) calculations, such as in the case of the DES5M set with five million dimers, they used a machine learning approach combined with MP2, SNS-MP2, which was found to have good agreement with CCSD(T). The SPICE (Small-Molecule/Protein Interaction Chemical Energies) dataset includes energies calculated at the ωB97M-D3BJ/def2-TZVPPD theory level for over 100,000 molecules as a resource for training machine learning potentials; it does not include nucleic acids [38]. The BEGDB database includes energies for hundreds of molecules and complexes calculated at various theory levels, with CCSD(T) and CBS-CCSD(T) results used as the benchmark to evaluate the performance of other methods [45]. BEGDB mainly focused on peptides studying the non-covalent interactions governing the biomolecules such as hydrogen bonds, dispersion interactions, and halogen binding.

Adenine dimer base stacking interactions in both parallel and twisted conformers were studied with varying DFT methods (B97D, ωB97XD, M06-2X, and CAM-B3LYP) and basis sets (6-311++G(d,p), aug-cc-pVDZ, aug-cc-pVTZ, daug-cc-pVDZ, and def2-TZVPP). Starting structures were taken from work by Hobza et al. and optimized using Gaussian09 [46]. Of the method and basis set combinations used, the recommended methods were ωB97XD/6-311++G(d,p), ωB97XD/aug-cc-pVDZ, and B97D3/aug-cc-pVDZ. Optimization with CAM-B3LYP/def2-TZVPP sometimes resulted in unexpected geometries and was thus not recommended for studying π-stacking interactions. Optimizations with aug-cc-pVTZ and daug-cc-pVDZ basis sets had difficulty reaching convergence for some structures and were also not recommended. The relationship of the energetic components of base stacking interactions obtained using the density-fitting-wave-function-based DF/SAPT based on SAPT0/jun-cc-pVDZ to the helical parameters of Rise, Twist, and Slide were studied, along with additional distributed multipole analysis (DMA) at HF/6-311G** to further elucidate the role of charge penetration [47]. Dispersion was the component that changed the most with Twist at low Rise; electrostatics changed the most at high Rise. Dispersion was the dominant attractive term overall, but electrostatics became highly attractive at low Rise due to charge penetration effects.

Comparisons between fixed charge MM and (fairly) large-scale QM methods in describing fairly large nucleic acid-containing systems have been made [48,49]. One study compared the description of the 11 nucleotide Sarcin-Ricin RNA motif by several QM methods and with AMBER, all with implicit solvent. The results from TPSS-D3/def2-TZVP, PW6B95-D3/def2-TZVP, and M06-2X were consistent; TPSS-gCP-D3/def2-SVP was also similar. PM6-D3H and HF-3c were not recommended. Overall, MD simulations were not shown to reproduce the native conformation; the inclusion of specific water molecules was only somewhat successful in amending this [48]. Another study compared the description of protein-nucleic acid interactions in a truncated MutS system (170 amino acid residues and 30 nucleic acids) by QM (FMO2-DFTB3/D3(BJ)/3ob theory level) and MM with AMBER, including explicit solvent molecules in the MD simulations. Interactions between backbone and functional groups of both the protein and nucleic acid were investigated, including energy decomposition with fragment molecular orbital (FMO). As expected, MM with the fixed charge model did not accurately capture dispersion or charge transfer. The MM results showed a larger departure from QM with the inclusion of solvent effects. As the MM model employed fixed charges, solvent screening effects were not included; this resulted in the repulsion overcoming the attraction for the A:T and G:C base pairs [49].

The interaction energies of base-paired and stacked nucleobases were also studied using various QM methods and compared to CBS/CCSD(T) and MM forcefields [25,50]. Natural base pairs were found to be more stable with longer hydrogen bond distances compared to the naturally occurring DNA base pairs [25,50]. MM electrostatics were found to perform best for the structures corresponding to the energy minima due to the cancellation of errors and to perform worst at short intermolecular distances [25,50].

In this work, we applied several QM methods to the non-covalent interactions of nucleic acid fragments after benchmarking against CCSD(T) on selected functional groups, similar to the work by Riley and Hobza [51]. As we will show, selected methods can be as accurate as the CCSD(T) but with much more tractable computational cost when applied to a large number of molecules and structures. In addition, SAPT and ALMO can decompose interaction energy into electrostatics, van der Waals, induction, etc., which is extremely valuable but not accessible through experiments. Multiple methods were used for each energy decomposition to ensure that results were robust. Such data regarding nucleic acids are lacking in the literature, which is the primary motivation of this work. Here, extensive calculations of nucleic acid interactions have been performed using SAPT0 with jun-cc-pVDZ, SAPT2+ with aug-cc-pVDZ, SAPT2+(3)/dmp2 with aug-cc-pVDZ, SAPT2+(3)/dmp2 with aug-cc-pVTZ, and ALMO EDA2 (original energy decomposition scheme) with B97M-V and aug-cc-pVTZ.

**Figure 1:**
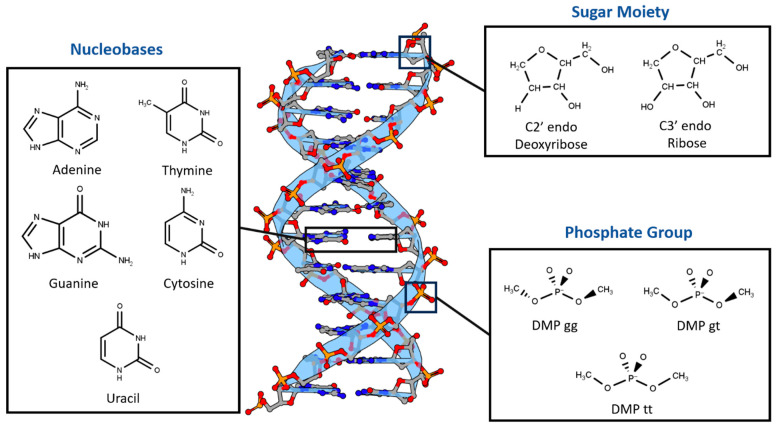
Nucleotides are dissected into three main constituents based on differences in chemical nature: Dimethyl Phosphate (DMP), Deoxyribose and Ribose Sugars, and Nucleobases. DNA representative structure is generated using 3D Protein Imaging [53].

Nucleotides were divided into three main components based on differences in chemical structure and charge (Figure 1). First, two sugar moieties were selected: neutral C2′-endo deoxyribose, the most common in B-DNA, and neutral C3′-endo ribose, the most common in both RNA and A-DNA. Second, the three most probable configurations of the negatively charged dimethyl phosphate were chosen: DMP gg (gauche-gauche), DMP gt (gauche-trans), and DMP tt (trans-trans). The main non-covalent interaction that governs the bonding of the last two moieties is the hydrogen bond. The third nucleotide component we investigated was the nucleobases, which interact via both hydrogen bonds and stacking interactions. The interaction of the three main components of the nucleotides with a water molecule over a wide range of locations and distances was studied to probe the hydrogen bond interaction aspect of the nucleotides. Additionally, stacked nucleobases (intrastrand) and hydrogen-bonding nucleobases (interstrand) were examined. Similar fragments, including nucleobases interacting with water and with each other via hydrogen bonding and π-stacking in pairs, have previously been used to build a neural network that was able to produce accurate electrostatic potentials for double-stranded DNA [52].

ALMO EDA2 was found to be consistent among selected DFT methods and independent of the basis sets used. On the other hand, the three levels of SAPT investigated (SAPT0, SAPT2+, and SAPT2+(3)/dmp2) showed high dependence on the basis set used. Upon studying the interactions of the intrastrand stacked nucleobases, interstrand nucleobases, and the three main fragments with water and nucleobases, the two of the best ab initio methods for the nucleotides are ALMO EDA2 (B97M-V) with aug-cc-pVTZ basis set and SAPT2+(3)/dmp2 with aug-cc-pVTZ.

Examination of the energy components of the interactions among the various constituents of nucleotides showed that exchange-repulsion was dominate within the neutral sugar moiety. The electrostatic attraction was also strong, largely offsetting the repulsion. Similar trends were observed for interactions of neutral nucleobases with water. As expected, electrostatics were more significant in the charged DMP interaction with water molecules. In all these cases, there is a large cancellation between the electrostatic attraction and exchange-repulsion. As a result, the attractive induction and dispersion are responsible for stabilizing the molecular complexes with water, highlighting the importance of explicit consideration of the induction effect. For intrastrand π-stacking nucleobase dimers, dispersion was found to be the dominant component, counterbalanced partially by repulsion. For interstrand hydrogen-bonding nucleobase pairs, repulsion was smaller. For G:C, attractive electrostatics were the dominant component; for A:T, dispersion was the dominant stabilizing component. This preliminary investigation of what methods perform the best in describing these nucleic acid fragments and which energetic components are likely to be important can serve as the basis for further QM study with the goal of developing more accurate polarizable force field (AMOEBA) models for nucleic acids.

## 2. Results

### 2.1. Small Molecule Benchmarking

#### 2.1.1. Various Theoretical Methods

Nucleotides mainly participate in two types of interactions: stacking interactions (between the nucleobases) and hydrogen bond interactions (involving nucleobases, sugar moieties, phosphate groups, and water solvent molecules). To determine the best available theoretical method for studying the interaction energies in such a complex system, benchmarking was performed on six hydrogen-bond forming compounds: Ethylamine-water (CCN-Wat), Methanethiol-Water (CS-Wat), Ethylamine-Methanethiol (CCN-CS), Methanol-Water (CO-Wat), Methanol-Ethanethiol (CCS-CO), and Chlorobenzene-Ethylamine (BenCl-CCN) and 6 stacked molecular dimers: Pyrrole–Pyrrole, Benzene-Imidazole, Benzene-Benzene, Fluorobenzene-Fluorobenzene, Fluorobenzene-Imidazole, Chlorobenzene-Chlorobenzene. These dimers were chosen as they were most chemically similar to nucleobases among those reported in the DE Shaw database, which includes interaction energies at the MP2 (Møller-Plesset perturbation), CCSD(T) Coupled Cluster Theory through perturbative triples, and Complete Basis Set CBS-CCSD(T) theory levels [44]. Here, we compared these methods with (1) DFT-based Absolutely Localized Molecular Orbitals Energy Decomposition Analysis (ALMO EDA2) with the original and the new energy decomposition schemes [28,29,30,54] and (2) different theory levels of Symmetry-Adapted Perturbation Theory (SAPT): SAPT0, SAPT2+, and SAPT2+(3)/dmp2 [27,55,56]. The aug-cc-pVTZ basis set was used for both ALMO and SAPT calculations. These methods also offer valuable interaction energy components that aid in understanding the driving forces for intra- and intermolecular complexes and developing accurate atomic potentials for molecular simulations. The most accurate method here, CBS-CCSD(T), was used as the benchmark.

**Figure 2:**
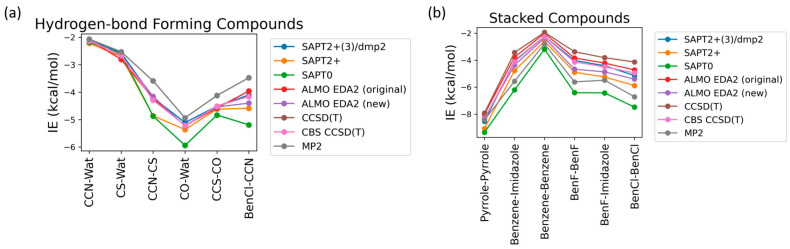
Benchmarking of the interaction energies of small molecules using various theoretical methods, mainly different levels of SAPT (SAPT0, SAPT2+, and SAPT2+(3)/dmp2) and ALMO EDA2 (B97M-V) with original and new energy decomposition schemes against CBS-CCSD(T). All the calculations were performed using the aug-cc-pVTZ basis set. (**a**) Interaction energies of hydrogen-bond forming compounds; (**b**) pi-stacking compounds.

The interaction energies of the hydrogen-bond-forming compounds from the different methods were similar, with the most deviation observed in the SAPT0 and MP2 methods (Figure 2a). The MP2 method was found to underestimate the interaction energies compared to CBS-CCSD(T), with the largest difference of 0.7 kcal/mol observed for the CCN-CS dimer interaction energy. SAPT0 overestimated the interaction energies, with the biggest deviation from CBS-CCSD(T), −1.0 kcal/mol, for BenCl-CCN. SAPT2+ performed well for most of the hydrogen-bond forming dimers except for CCN-CS, where the difference from CBS-CCSD(T) was −0.57 kcal/mol. In summary, all of the tested methods showed relatively small deviations compared to CBS CCSD(T). The best performance was given by SAPT2+(3)/dmp2 and ALMO EDA2 with both old and new energy decomposition schemes—the maximum deviation from CBS CCSD(T) was 0.1 kcal/mol (Figure 2a).

The interaction energies obtained from the various theoretical methods were found to vary much more significantly for the stacked complexes (Figure 2b) compared to the hydrogen-bond forming complexes. CCSD(T), MP2, SAPT0, and SAPT2+ overestimated the interaction energies compared to CBS-CCSD(T), with the most deviation observed for SAPT0. The interaction energy of the BenCl–BenCl dimer obtained from the various methods was found to be associated with the maximum difference compared to CBS-CCSD(T) interaction energies. For example, the SAPT0 interaction energy deviated from the CBS-CCSD(T) value by 2.54 kcal/mol; for the MP2 method, the deviation was 1.77 kcal/mol. BenF–BenF and BenF–Imidazole interaction energies also showed large variations among different methods. This showcases the limitation of these methods with the halogen-substituted aromatic systems in general. As shown in Figure 2b, the theoretical methods with the least deviation from CBS-CCSD(T) for the stacked molecules were found to be SAPT2+(3)/dmp2 (maximum of 0.2 kcal/mol observed in BenCl–BenCl), followed by ALMO EDA2 with the original energy decomposition (maximum deviation of 0.36 kcal/mol observed in BenCl–BenCl), and then ALMO EDA2 with the new energy decomposition (maximum difference of 0.54 kcal/mol observed in BenF–BenF).

#### 2.1.2. Comparison of DFT Methods

**Figure 3:**
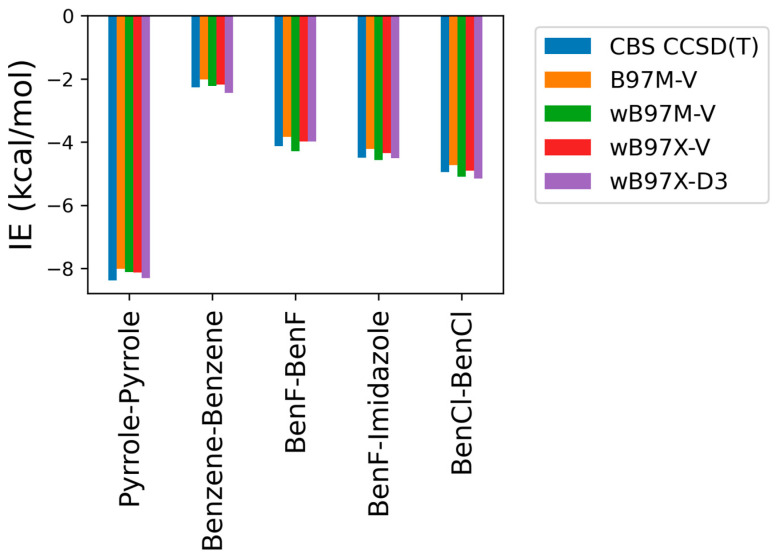
Interaction energies for the π-stacking molecules obtained using ALMO EDA2 with different levels of DFT theory (B97M-V, ωB97M-V, ωB97X-V, and ωB97X-D3) compared to the CBS-CCSD(T) benchmark.

As some of the tested theoretical methods were inconsistent in the case of the stacked complexes compared to the hydrogen-bond forming complexes, further analysis was performed on the stacked compounds using different DFT levels of theory with ALMO EDA2. Four different DFT methods (B97M-V, ωB97M-V, ωB97X-V, and ωB97X-D3) were tested and contrasted with CBS-CCSD(T). This additional comparison was performed due to the superior performance of both the original and the new energy decomposition schemes of ALMO EDA2, based on DFT calculation, for the hydrogen bonding and stacked complexes. The interaction energies obtained from the four tested DFT levels of theory were found to be very similar to each other and to CBS-CCSD(T) interaction energies for the stacked molecule (Figure 3). B97M-V showed the most deviation from the CBS-CCSD(T) method, yet the maximum error (pyrrole–pyrrole) was only 0.36 kcal/mol. Interestingly, pyrrole–pyrrole was the dimer showing the largest variation among the different DFT methods, except for ωB97X-D3, where the largest error was associated with BenCl–BenCl. The ωB97X-D3 method yielded the results most consistent with CBS-CCSD(T), with a maximum difference of 0.21 kcal/mol among the test sets. Overall, the results indicate that all these DFT methods tested here produce satisfying interaction energies that closely match the CBS-CCSD(T) results (Figure 3).

#### 2.1.3. Basis Set Dependence

**Figure 4:**
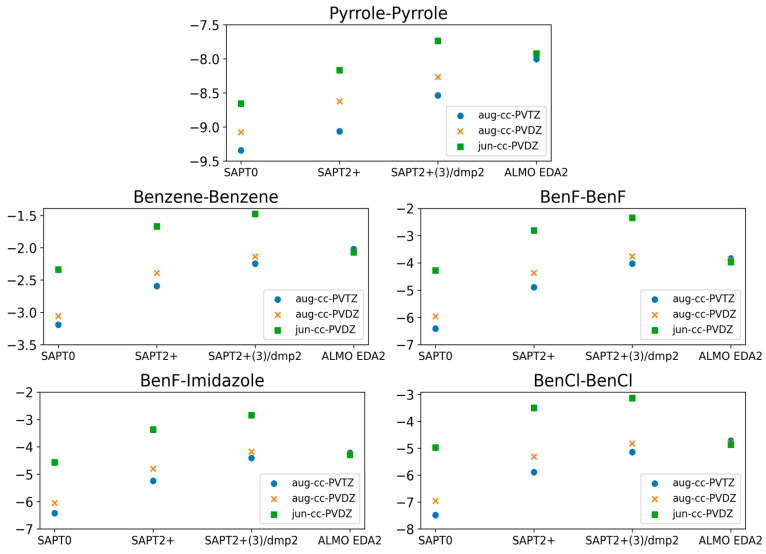
Comparison of the interaction energies of the stacked molecules calculated using ALMO EDA2 with B97M-V, SAPT0, SAPT2+, and SAPT2+(3)/dmp2 with three different basis sets (jun-cc-PVDZ, aug-cc-PVDZ, and aug-cc-PVTZ). The ALMO EDA2 results did not display significant basis set dependence. It was determined that SAPT0 with jun-cc-pVDZ basis and SAPT2+ and SAPT2+(3)/dmp2 with aug-cc-pVDZ are in reasonable agreement with ALMO EDA2, with an average deviation of 0.2 kcal/mol for all stacking energies. The maximum difference among the three SAPT methods was 0.3 kcal/mol, observed for BenCl–BenCl. SAPT0 showed better agreement with the smaller basis set, jun-cc-pVDZ, rather than showing accuracy increasing with basis set size.

The calculations showing superior performance of ALMO EDA2 (B97M-V) with original energy decomposition scheme and SAPT2+(3)/dmp2 methods for both stacked and hydrogen-bond forming compounds were performed using the aug-cc-pVTZ basis set. Benchmarking of the basis set dependence of both ALMO and SAPT methods is also crucial. Three different basis sets (aug-cc-PVTZ, aug-cc-PVDZ, and jun-cc-PVDZ) were used for the five stacked molecules using different levels of theory: SAPT0, SAPT2+, SAPT2+(3)/dmp2, and ALMO EDA2 (Figure 4).

The ALMO EDA2 results showed independence from the basis set used; the maximum deviation in the interaction energies for all five stacked molecules calculated using the different basis sets was ~0.1 kcal/mol. This reflects the robustness of the DFT methods such as B97M-V. The situation with SAPT was more complicated. Using the larger basis sets (aug-cc-pVTZ and aug-cc-pVDZ) with SAPT0 resulted in an overestimation of the interaction energy by 3.0 kcal/mol on average, especially for halogen substituted benzene molecules, compared to SAPT0 with the smallest basis set jun-cc-pVDZ. While we would have expected higher theory levels and larger basis sets to result in more accurate energies, SAPT0 with jun-cc-pVDZ resulted in interaction energies that were more consistent with those obtained from ALMO EDA2. Interestingly, SAPT0 with jun-cc-pVDZ has been shown previously to reasonably describe stacking energies in the literature [47,56,57]. In higher theory levels of SAPT (SAPT2+ and SAPT2+(3)/dmp2), using jun-cc-pVDZ resulted in an underestimation of the interaction energy by ~2.0 kcal/mol for the five stacked dimers. The interaction energies obtained using SAPT2+/aug-cc-pVDZ were closer to those obtained using ALMO aug-cc-pVDZ than those obtained using SAPT2+/aug-cc-pVTZ. The maximum difference in energies obtained using the two basis sets was ~0.58 kcal/mol, observed for BenCl–BenCl. The performance of SAPT2+(3)/dmp2 with aug-cc-pVDZ was slightly closer to the ALMO energies than SAPT2+(3)/dmp2 with aug-cc-pVTZ. The difference between energies calculated with the two basis sets (aug-cc-pVTZ and aug-cc-pVDZ) at the highest level of theory (SAPT2+(3)/dmp2) was found to be smaller than it was with SAPT2+.

### 2.2. Total Interaction Energies of the Main Three Nucleotide Constituents

Based on the benchmarking results so far, we applied the following five protocols to examine the interaction energies of nucleotide fragments: ALMO EDA2 (B97M-V, original scheme), SAPT0 with jun-cc-pVDZ, SAPT2+ with aug-cc-pVDZ, and SAPT2+(3)/dmp2 with either aug-cc-pVTZ or aug-cc-pVDZ.

#### 2.2.1. Sugar Moiety

**Figure 5:**
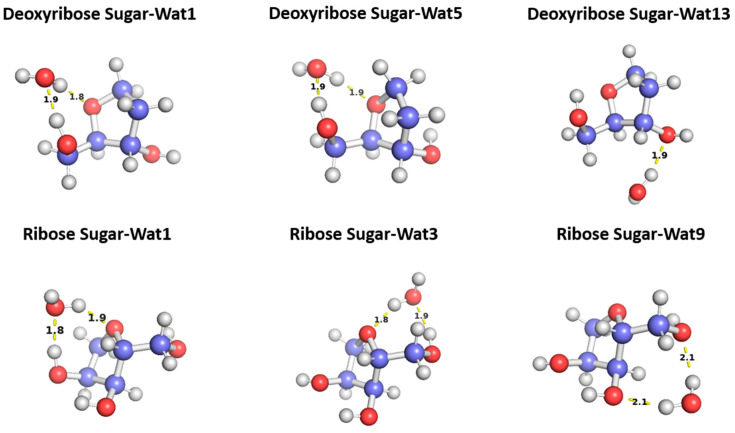
Representative structures for the C2′-endo deoxyribose sugar and C3′-endo ribose sugar interacting with water probes at different positions. The distances correspond to the equilibrium distance obtained from MP2 optimization of the structures with a 6-31G* basis.

To probe the hydrogen binding interaction around the sugar moiety, three different configurations of a water molecule interacting with C2′-endo deoxyribose and three of water interacting with C3′-endo ribose sugars were selected based on the optimal docking scores obtained from the aISS workflow [58] and on the uniqueness of the water position (Figure 5). To probe the interaction energy surface, the water probe was moved away from the minimum energy distance along the vector connecting the two molecular centers of mass. For each distance, the single-point interaction energy of sugar-water was calculated.

**Figure 6:**
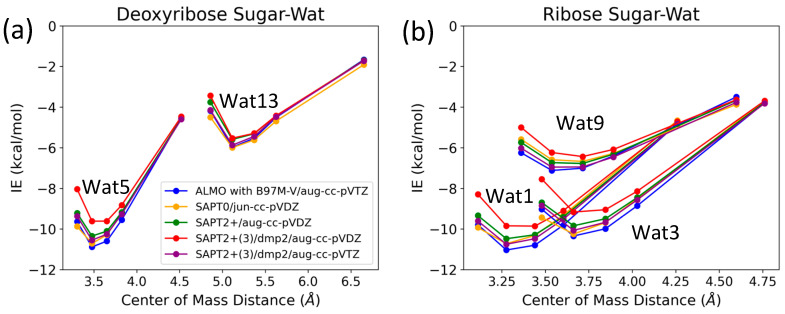
Interaction energy curves of (**a**) C2′-endo deoxyribose sugar and (**b**) C3-endo ribose sugar with different positions of water molecule at 0.95, 1.0, 1.0, 1.05, 1.1, and 1.3 times the separation distance between the centers of mass at equilibrium.

The three configurations of water interacting with deoxyribose chosen were Wat1, Wat5, and Wat13 (Figure 5). In the Deoxyribose-Wat1 and Wat5 configurations, the water molecules form hydrogen bonds with the oxygen of the five-membered ring sugar moiety and with the terminal hydroxyl group. These configurations differ in the angle of interaction with the sugar moiety but have similar interaction distances of 1.8 or 1.9 Å. The Wat13 configuration includes the formation of a single hydrogen bond with the hydroxyl group attached to C3′ at 1.9 Å. The Deoxyribose-Wat1 and Wat5 configurations were found to have lower energy compared to the Wat13 configuration due to the additional hydrogen bond formed. The Wat5 configuration had a slightly lower energy than the Wat1 configuration by about 0.6 kcal/mol. For clarity, only the Wat1 and Wat13 energy profiles are shown in Figure 6a.

Similarly, three different configurations of the water molecule interacting with C3′-endo ribose sugar moiety were selected (Figure 5). In the Wat1 configuration, the water molecule forms hydrogen bonds with the oxygen of the five-membered ring and the hydroxyl group attached to the C3′. In the Wat3 configuration, water forms hydrogen bonds with the oxygen in the ring and the terminal hydroxyl group. Finally, in the Wat9 configuration, hydrogen bonds are formed between water and the terminal hydroxyl group, as well as the hydroxyl group attached to C2′. The Ribose-Wat1 configuration was associated with the lowest energy, followed by the Wat3 and then Wat9 configurations. This indicates that the formation of a hydrogen bond with the oxygen of the ring stabilizes the complexes to the lowest energy configurations.

SAPT2+(3)/dmp2 with aug-cc-pVDZ resulted in the most inconsistent interaction energies compared to the other methods, with an average deviation of 1.2 kcal/mol at the equilibrium distance for all three configurations. This suggests that SAPT2+(3)/dmp2 with aug-cc-pVDZ should not be recommended for QM benchmarks for nucleic acids, even though the stacking calculations above indicated this protocol was “reasonable”. Similarly to the deoxyribose-water interaction curve, SAPT0 with jun-cc-pVDZ, SAPT2+(3)/dmp2 with aug-cc-pVTZ, and ALMO EDA2 second generation were found to be very consistent in the predicted interaction energies, except for the Wat9 configuration (Figure 6b). The stabilization energy is about −6 or −7 kcal/mol for a single hydrogen bond and about −11 kcal/mol for double hydrogen bonds SAPT2+ with aug-cc-pVDZ was found to be more consistent with the other protocols compared to SAPT0 for the wat9 configuration, with an average deviation of 0.2 kcal/mol between the two protocols for the different distances.

**Figure 7:**
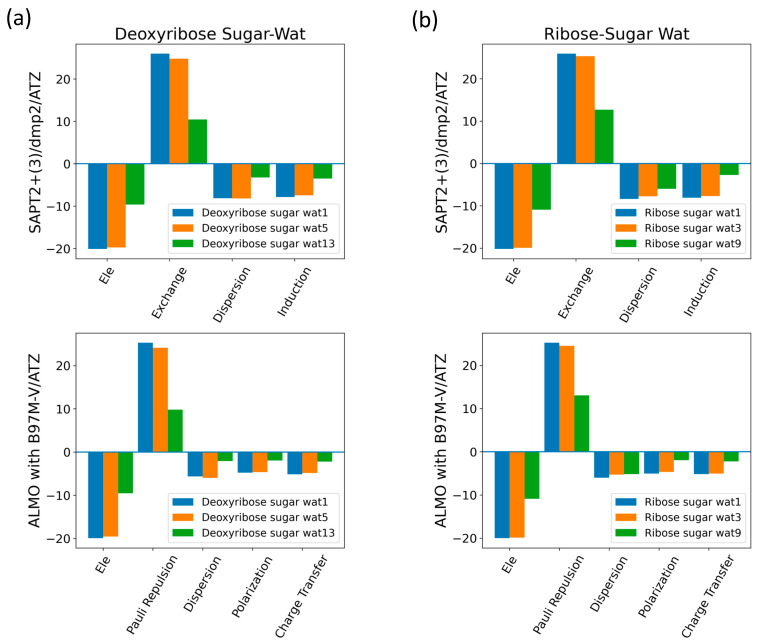
Energy decomposition using SAPT2+(3)/dmp2 and ALMO EDA2 for several configurations of water interacting with (**a**) C2′-endo deoxyribose and (**b**) C3′-endo ribose.

The interaction energy components were obtained from SAPT2+(3)/dmp2 and ALMO EDA2 (original scheme) with B97M-V aug-cc-pVTZ (ATZ) for the equilibrium structures of deoxyribose and ribose sugar moieties with the water probe (Figure 7). Overall, the EDA results from SAPT and ALMO are quite consistent with each other. The exchange repulsion was the most dominant positive component. Typically, the repulsion was ~25 kcal/mol for double h-bond complexes and ~10 kcal/mol for a single h-bond complex. The electrostatic, dispersion, and induction (polarization + charge transfer in ALMO) interactions comprise the attractive contributions to the binding energy. Electrostatics are clearly the major stabilizing force, contributing ~−20 kcal/mol in double h-bond complexes and ~−9 kcal/mol in single h-bond complexes. The separation between the dispersion and induction components differs slightly between SAPT and ALMO. Using SAPT, the dispersion is about −7 and −3 kcal/mol for double and single h-bond complexes, respectively. With ALMO, the dispersion is about 1–2 kcal/mol less negative than that of SAPT for both. However, the sum of polarization and charge transfer in ALMO is slightly more negative than the induction in SAPT but a similar amount. Thus, the total interaction energies are still consistent. Both EDA methods show that induction is significant, amounting to ~30–40% of the electrostatic energy. This again highlights the importance of explicit consideration of electronic polarization in classical force fields for the description of nucleic acids.

#### 2.2.2. DMP

**Figure 8:**
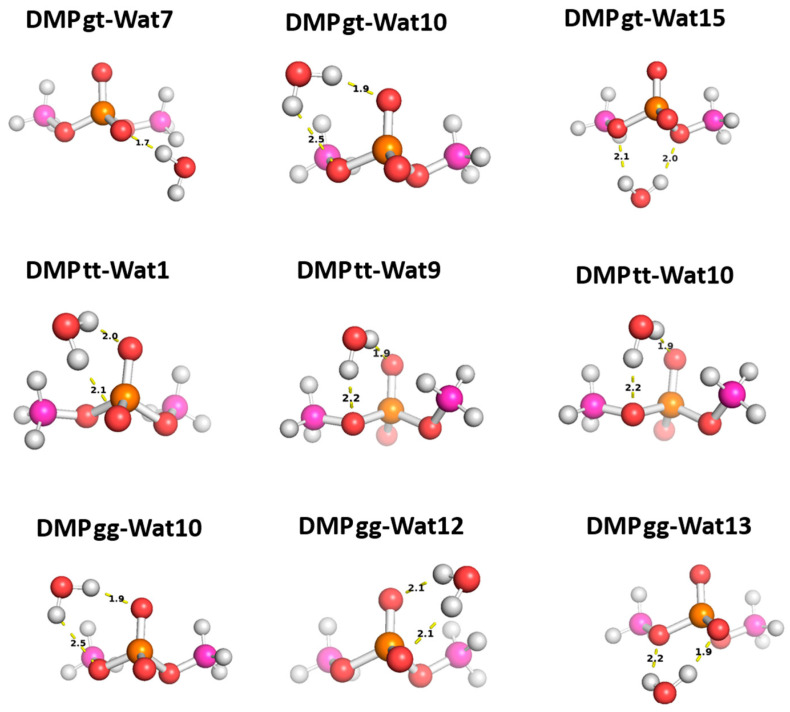
Optimized structures of the three dominant conformations of DMP [17]: DMP gg (gauche–gauche), DMP gt (gauche–trans), and DMP tt (trans–trans) interacting with water molecules in various positions. The distances between DMP and water shown correspond to equilibrium distances retrieved from MP2/6-31G* optimization.

Three different positions for the water molecules surrounding the low energy configurations of DMP [17] were selected to probe the functional groups of the DMP molecule: gauche–gauche (gg), gauche–trans (gt), and trans–trans (tt). In the Wat1-DMP tt configuration, the water molecule forms two hydrogen bonds with the phosphate group. In the Wat9 and Wat10 configurations, the water molecule forms a single hydrogen bond with one of the oxygen atoms of the phosphate group and another with the oxygen of the –OCH_3_ group attached to the phosphate (Figure 8). With the DMP gt configuration, water forms a single hydrogen bond with the oxygen atom of the phosphate group in the Wat7 configuration, one hydrogen bond with the phosphate group and one with the oxygen of the –OCH_3_ group in Wat10, and two hydrogen bonds with the oxygens of the two –OCH_3_ groups attached to the phosphate group in Wat15 (Figure 8). In the Wat10 DMP gg configuration, the water molecule forms a hydrogen bond with the phosphate group and with the oxygen of the –OCH_3_ group attached to it; the Wat12 and Wat13 configurations show the formation of two hydrogen bonds with the phosphate group (Figure 8).

**Figure 9:**
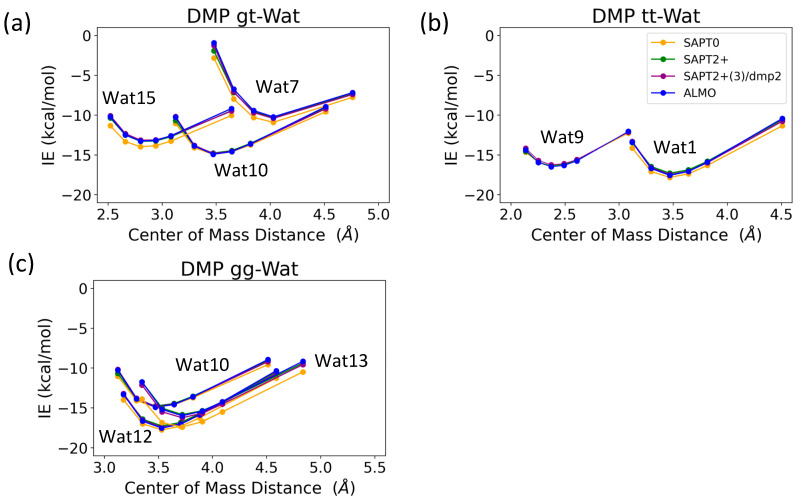
Energies for water in various positions interacting with (**a**) DMP tt, (**b**) DMP gt, and (**c**) DMP gg DMP at 0.9, 0.95, 1.0, 1.0, 1.0, 1.0, 1.05, 1.1, and 1.3 times the COM distance at equilibrium geometries.

The interaction energy of the DMP tt-Wat1 configuration was found to be the most favorable among the three configurations of DMP tt-water, followed by the Wat9 and Wat10 configurations, which had similar interaction energies (Figure 9). For clarity, Wat10 profiles are not shown here, as they largely overlap with those of Wat9. The two hydrogen bonds involving the two charged oxygen atoms in the phosphate group in the DMP tt-Wat1 configuration resulted in an interaction energy higher by ~−2.0 kcal/mol near the equilibrium distances compared to those where a hydrogen bond is formed with the sp^3^ oxygen of the phosphate group. Wat10 and Wat15 in the DMP gt configuration followed a similar trend, except that the interaction energy was slightly less favorable than those in the DMP tt conformation. DMP gt-Wat7 contains only one hydrogen bond; the interaction energy is much weaker than expected. Water interaction energies with the DMP gg conformer were generally similar to those observed for the DMP gt conformation (Figure 9). This suggests that the interaction of DMP with water has a nonnegligible conformational dependence, with the tt conformation being preferred. Again, the interaction energies were consistent across the tested QM methods, with the exception of the SAPT0/jun-cc-pVDZ basis set (Figure 9). Therefore, SAPT0 with jun-cc-pVDZ is not recommended as a general method for evaluating nonvalent interactions.

**Figure 10:**
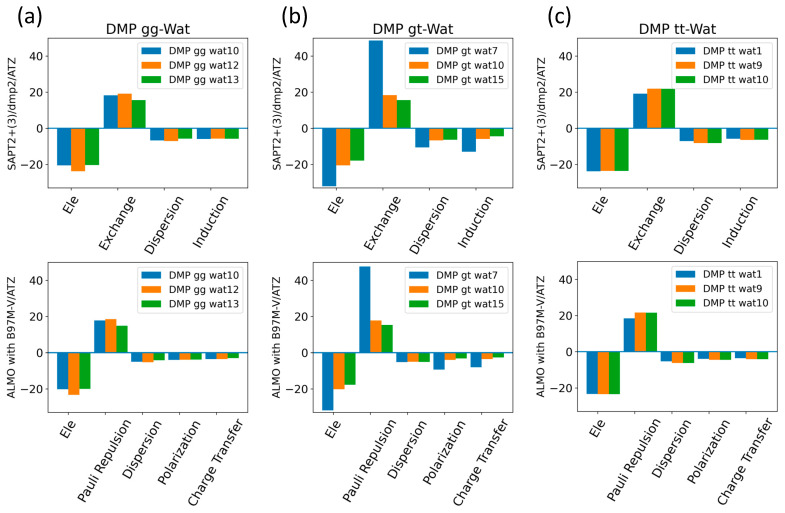
Energy decomposition using SAPT2+(3)/dmp2 and ALMO EDA2 for (**a**) DMP gg-Wat, (**b**) DMP gt-Wat, and (**c**) DMP tt-Wat interactions.

The EDA of the interaction of the negatively charged DMP gg with the water molecule at the equilibrium distance showed that the attractive electrostatic component is counterbalanced with the positive exchange-repulsion, similar to what was observed for sugar-water (Figure 10a). The main difference lies in the electrostatic component. A similar pattern was observed in the ALMO energy decomposition, wherein the dominant electrostatics component, with an average contribution of −22 kcal/mol for all three configurations, was counterbalanced by the Pauli repulsion, which averaged ~17 kcal/mol for the three configurations (Figure 10a). Electrostatic components were found to be dominant in both DMP gt-Wat and DMP tt-Wat configurations, with average contributions of −23.5 kcal/mol and −23.7 kcal/mol with SAPT2+(3)/dmp2, respectively, and an average of −23.3 kcal/mol for both with the ALMO energy decomposition (Figure 10b,c). DMP gt 7 was the only exception; the exchange component had the largest contribution to the total energy (48.5 kcal/mol), followed by the electrostatics component (−32.1 kcal/mol), as shown in Figure 10b. Also, the contribution of the induction and dispersion in DMP gt-Wat7 in SAPT2+(3)/dmp2 was found to be double that of the remaining configurations. This was similar to what was observed in the ALMO energy decomposition, where polarization and charge transfer were also twice as high as in the remaining configurations (Figure 10b). This large contribution to the polarization and energy transfer may be due to the small distance (1.7 Å) between water and the oxygen of the phosphate group of the DMP gt molecule, compared to 1.9 Å in DMP gt-Wat10 (Figure 8). For configurations other than the DMP gt-Wat7, the contribution of the induction and dispersion components in the SAPT2+(3)/dmp2 decomposition was smaller compared to the electrostatic and exchange components. This was also observed in the ALMO energy decomposition; the dispersion, polarization, and charge transfer components contributed less than the electrostatics and the Pauli repulsion for all DMP configurations (Figure 10).

#### 2.2.3. Nucleobases

##### Nucleobase-Water Interactions

**Figure 11:**
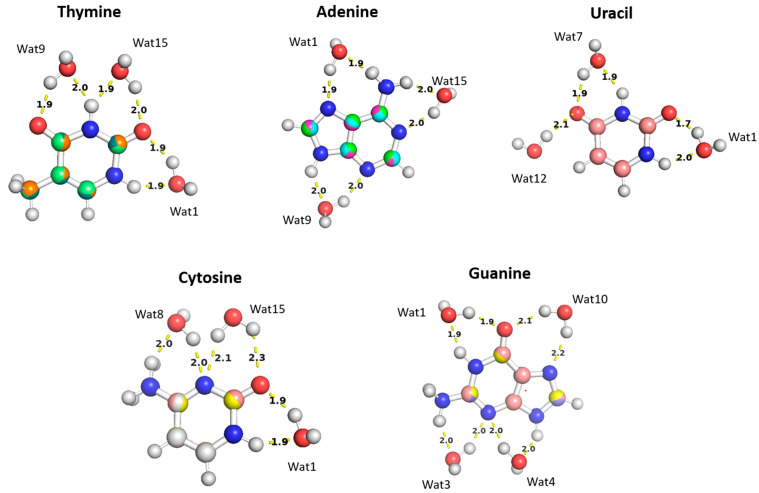
The optimized structures of the nucleobases (A, G, U, T, and C) interacting with water molecules in three different positions overlayed on top of each other. The distances shown correspond to the equilibrium distances at equilibrium retrieved from MP2/6-31G* optimization.

The aISS workflow [58] was used to generate 15 different conformations of purine (A and G) and pyrimidine (U, T, and C) nucleobases interacting with water probes at different positions. Three different positions of the water molecule probe around the A, U, T, and C nucleobases and four positions around the G nucleobase were selected in order to cover the interactions of the water molecule with all of the corresponding polarizable groups of the nucleobases (Figure 11). Optimization of the selected nucleobase-water structures was performed at the MP2/6-31G* theory level to obtain the equilibrium distances of the interaction of the nucleobases with the water probe at various positions.

The water molecules interacting with the Thymine nucleobase formed two hydrogen bonds, one with the hydrogen atom of the amine group and the other with the oxygen of the carbonyl group, at different positions and at various distances. In the Thymine-Wat1 (T-Wat1) configuration, water interacts with N1 (1.9 Å) and oxygen at C2 (1.9 Å). In T-Wat9, it interacts with N3 (2.0 Å) and oxygen at C4 (1.9 Å). Finally, the water in T-Wat15 interacts with N3 (1.9 Å) and the oxygen atom at C2 (2.0 Å).

The water in Adenine-Wat1 (A-Wat1) interacts with the terminal NH_2_ group and N7 of the purine ring at 1.9 Å. In the A-Wat9 configuration, water interacts with N3 and N9 at an equal distance of 2.0 Å. In A-Wat15, it interacts with N1 of the purine ring and the terminal NH_2_ group at 2.0 Å. Uracil-Wat1 and U-Wat7 show similar interaction patterns, wherein Wat1 interacts with N1 and with the carbonyl group at C2 at 1.9 Å and 1.7 Å, respectively. The water of U-Wat7 interacts with both N3 and the carbonyl of C4 at 1.9 Å. On the other hand, U-Wat12 forms a single hydrogen bond with the carbonyl group of C4.

Water molecules at all positions interact with the Cytosine nucleobase with the same interaction pattern, forming two hydrogen bonds with the hydrogen of NH_2_ and the oxygen of the carbonyl group at different positions. The water in C-Wat1 interacts with N1 and the oxygen atom of the carbonyl group at C2. In C-Wat9, water interacts with N3 and the oxygen of the carbonyl group C4. Finally, Wat15 interacts with N3 and the oxygen atom of the carbonyl group at C2 in the C-Wat15 configuration.

Four water probes were needed to cover all of the polarizable groups of the Guanine nucleobase. G-Wat1 and G-Wat10 showed similar interaction patterns to each other and to the other water—nucleobase pairs. Both Wat1 (hydrogen atom of N1 and oxygen atom at carbonyl group of C6) and Wat10 (N7 oxygen atom at carbonyl group of C6) interact with an amine group and with the oxygen atom of the carbonyl group. The G-Wat3 and G-Wat4 waters interact with amine groups at equal distances of 2 Å with N3 and NH_2_ attached at C2 and with N3 and N9, respectively (Figure 11). The water probe interacting with the nucleobases at different geometries was then translated along a vector between the center of mass of the two molecules to 0.90, 0.95, 1.0, 1.1, and 1.3 times the equilibrium distance. Energies were then calculated at each point to generate an interaction energy curve of the nucleobases with the water probes.

**Figure 12:**
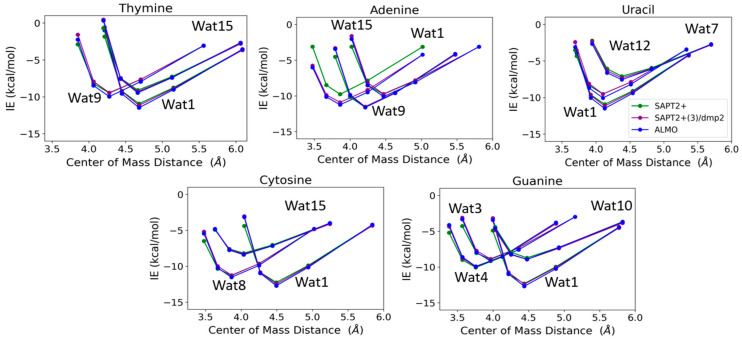
Interaction energies of the purine and pyrimidine nucleobases with water probes at various positions over distances between the centers of mass of the molecules varied to 0.9, 0.95, 1.0, 1.1, and 1.3 times that at the equilibrium geometry. The distances between the center of mass shown in the plot do not necessarily reflect the interaction distance between the water probes and the nucleobases.

T-Wat1 was found to have the lowest energy among the three conformations of T-Wat interacting complexes (Figure 12). This may be attributed to the shorter distance of the two hydrogen bonds formed by the Wat1 probe (1.9 Å) compared to the other two water probe positions (1.9 Å and 2.0 Å), as shown in Figure 11. For the Adenine-water interaction, A-Wat9 was found to have the lowest energy conformation, followed by A-Wat1. A-Wat15 was found to have the highest energy among the three conformations. The interaction of the Wat12 probe with Uracil was found to be the least energetically stable U-Wat conformation; it forms a single hydrogen bond compared to Wat1 and Wat7, which form two hydrogen bonds (Figure 11). The Wat1 interaction with Uracil was more stable than with Wat7; the corresponding hydrogen bond interaction distance is shorter. In the case of Cystine, the C-Wat15 complex had the lowest interaction energies due to the large distances of the two formed hydrogen bonds at 2.1 Å and 2.3 Å, compared to C-Wat1 (1.9 Å) and C-Wat8 (2.0 Å) interaction energies.

G-Wat1 was found to have the lowest energy among the four Guanine-water conformations, with the shortest hydrogen bond distance (1.9 Å) in contrast with G-Wat10 (2.1 and 2.2 Å), which has the highest interaction energy despite having similar interactions involving oxygen and nitrogen in the ring. The G-Wat3 and G-Wat4 complexes were less favorable than G-Wat1 (Figure 12), likely due to the interaction with the hydrogen of -NH_2_ being weaker than the interaction with the oxygen of the carbonyl group. The Wat4 probe—which forms two hydrogen bonds with the amine of the Guanine ring—was found to have a higher interaction energy with Guanine than the Wat3 probe, which shows hydrogen bonds between water and one amine in the ring plus one terminal amine. This is likely due to stabilization due to resonance in the purine ring.

The SAPT2+(3)/dmp2 with aug-cc-pVTZ and ALMO EDA2 with B97M-V water-nucleobase interaction energies showed good agreement with each other (Figure 12). A maximum deviation of -1.2 kcal/mol between ALMO EDA2 generation and SAPT2+/aug-cc-pVDZ was observed at the shortest interaction distance between the Wat15 probe and Adenine (A-Wat15); RMSE was found to be 0.52. This reflects that SAPT2+/aug-cc-pVDZ may describe interactions at short distances more poorly than the other two methods. Because of this, we did not continue using this method as a possible QM benchmark to study the interaction energies of nucleic acid components.

**Figure 13:**
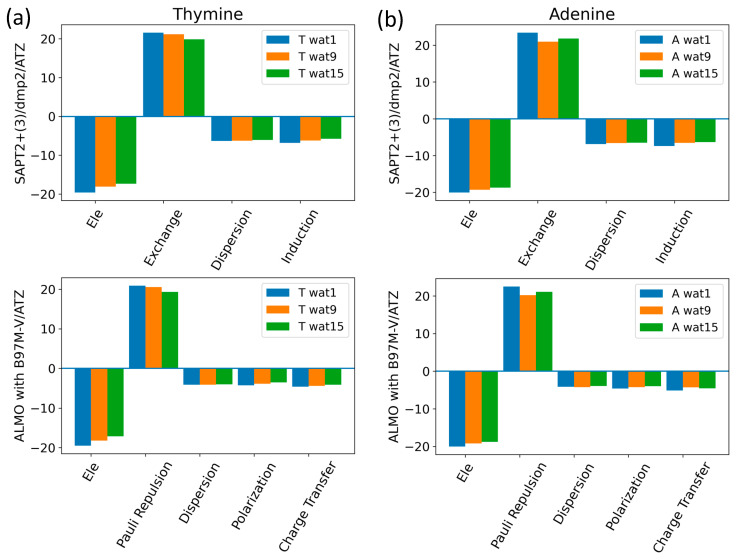
Analysis of total energy components using SAPT2+(3)/dmp2 and ALMO EDA2 for Nucleobase-water configurations. (**a**) Energy components of Thymine-Wat; (**b**) Energy components of Adenine-Wat.

The components of the total interaction energies of pyrimidines and purines with water were investigated using Thymine and Adenine, respectively (Figure 13). Similarly to what we saw for the interaction of water with the neutral sugar moiety (Figure 7), the dominant energetic contributions were exchanged with SAPT2+(3)/dmp2 and Pauli repulsion with ALMO EDA2, followed by the partially counterbalancing attractive electrostatic component. Dispersion and induction (polarization + charge transfer) were found to contribute to a lesser extent, with an average of −6.5 kcal/mol for each component in the different Nucleobase-Wat configurations. The difference in the purine and pyrimidine structures did not have a large impact on the contribution of the different energy components to the total interaction energy.

##### Intrastrand and Interstrand Nucleobases

**Figure 14:**
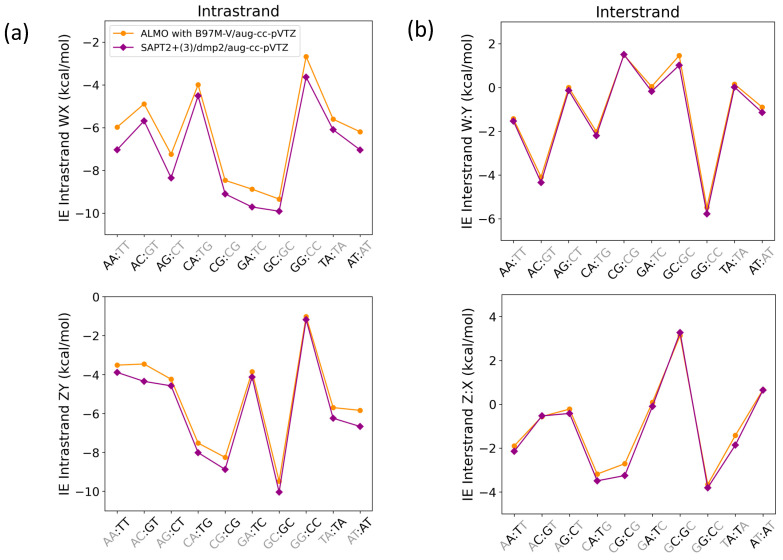
Interaction energies for base step pairs using SAPT2+(3)/dmp2 and ALMO with B97M-V for (**a**) Intrastrand nucleobases governed by π-stacking interactions and (**b**) interstrand nucleobases governed by a hydrogen bond. Each base step (WX:ZY) includes stacking (WX and ZY) and hydrogen bonding (W:Y and Z:X).

Nucleobase intrastrand π-stacking and interstrand hydrogen bonding were studied using SAPT2+(3)/dmp2 and ALMO EDA2 with B97M-V (Figure 14) with systems containing one base step (2 base pairs on each strand). The structures and respective nomenclature of the interacting nucleobases in the B-DNA (most common) form were obtained from work published by Sherill et al. [25]. While the energies from the two methods were similar for the interstrand interactions (Figure 14b), there were differences between them in the description of intrastrand stacking interaction energies (Figure 14a).

The average root-mean-square-error (RMSE) for both WX and ZY intrastrand nucleobases was 0.68, while the average RMSE for both W:Y and Z:X interstrand nucleobases was found to be 0.25. The maximum deviation in the intrastranded WX nucleobase interaction energies between SAPT2+(3)/dmp2 and ALMO/B97M-V was observed in the stacked AG (−1.11 kcal/mol), followed by stacked AA (−1.06 kcal/mol), shown in Figure 14a. The maximum deviation in the intrastrand ZY nucleobase interaction energies was observed for GT (−0.89 kcal/mol), followed by AT (−0.83 kcal/mol) (Figure 14a). The interstrand W:Y nucleobase interaction energies displayed a smaller deviation between the two methods. The largest difference of −0.25 kcal/mol was observed for GC of GC:GC, followed closely by −0.24 kcal/mol for A:T of AT:AT (Figure 14b). Of the Z:X interstranded nucleobase interactions, the largest deviation of −0.54 kcal/mol was observed for the G:C base pair of CG:CG.

**Figure 15:**
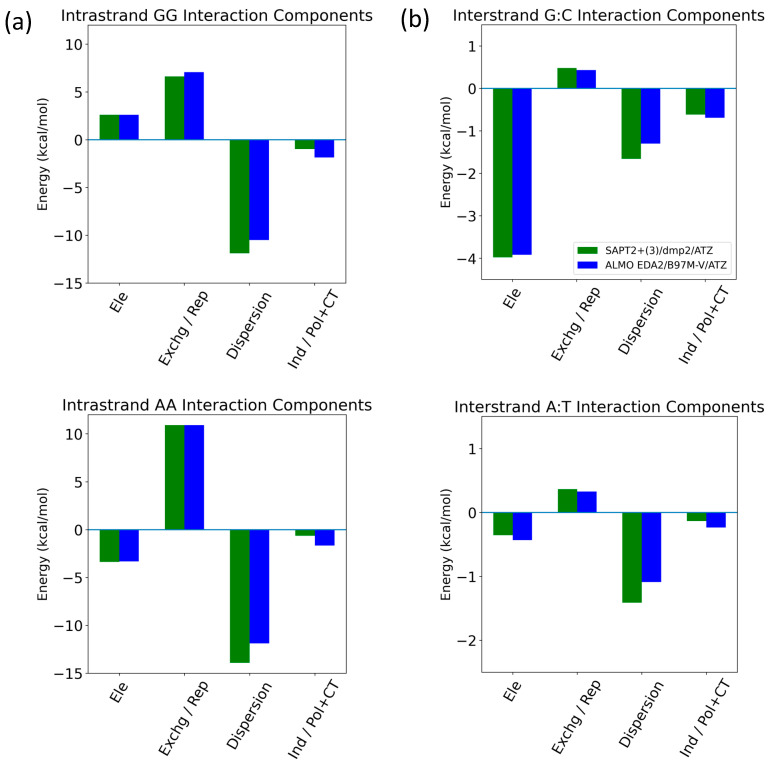
Decomposition of total energy components using SAPT2+(3)/dmp2 and ALMO EDA2 with B97MV into electrostatics (Ele), exchange (Exchg, SAPT)/Pauli Repulsion (Rep, ALMO), dispersion, and induction (Ind, SAPT)/polarization + charge transfer (Pol + CT, ALMO) for (**a**) intrastrand GG and AA base stacking and (**b**) interstrand G:C and A:T hydrogen bonding.

The energetic components of intrastrand GG/AA and interstrand G:C/A:T were analyzed as examples of π-stacking and hydrogen bonding interactions in the nucleic acid, respectively (Figure 15). For the intrastrand GG base stacking interaction, SAPT2+(3)/dmp2 and ALMO/B97M-V agreed that dispersion was favorable and the largest component, followed by the unfavorable exchange (SAPT)/Pauli Repulsion (ALMO). (Figure 15a) Electrostatics contributed to destabilization to a lesser extent. Induction (SAPT)/polarization + charge transfer (ALMO) added a small favorable contribution. For the intrastrand AA base stacking interaction, dispersion was again the dominant component, counterbalanced partially by exchange/repulsion. Electrostatics are once more the third largest component; however, they were attractive for AA, unlike what we saw for GG. Induction was again small and negative. For the interstrand G:C hydrogen bonding base pair, both methods showed that attractive electrostatics dominated, followed by dispersion and induction, which were also stabilizing (Figure 15b). While dispersion and electrostatics were still the two largest stabilizing energetic components for the A:T hydrogen bonding interaction, electrostatics were weaker, and dispersion was now the dominant component. Induction was smaller than repulsion for A:T, unlike the results for G:C, but still a stabilizing contribution. Exchange/repulsion was destabilizing but small for both A:T and G:C. The SAPT and ALMO methods differed most in the description of dispersion, especially for AA intrastrand base stacking. However, the overall agreement between the two methods lends robustness to the results.

## 3. Discussion

Nucleic acid plays a vital role in several biological processes, from contributing to the central dogma of the cell to functioning as an enzyme or binding as a ligand. With the current limitations of the MM forcefields in dealing with the non-covalent interactions dominating the nucleic acid, especially the π-stacking interactions due to the lack of charge penetration factor, an accurate description of the non-covalent interactions will help in developing better forcefields tailored for the nucleic acid. Small nucleotide benchmarking was done to evaluate the performance of several QM methods in describing the energetic components of hydrogen bonding and π-stacking interactions in aromatic compounds. These methods showed excellent agreement on the hydrogen bonding compounds, with SAPT2+(3)/dmp2 and ALMO EDA2 (old and new decomposition schemes) matching most closely with the gold standard CCSD(T) and CBS-CCSD(T) results. The stacking interaction results displayed more variation, but again, the best performance was by SAPT2+(3)/dmp2 and ALMO EDA2. It was previously reported that pure DFT functionals were unsatisfactory for base stacking interactions but performed better for hydrogen bonding.

The stacked molecules were then further investigated using ALMO EDA2 with varying DFT methods (B97M-V, ωB97M-V, ωB97X-V, and ωB97X-D3) and compared to CCSD(T), with very similar results for all. Next, possible basis set dependence was assessed by varying the basis set (aug-cc-pVTZ, aug-cc-pVDZ, jun-cc-pVDZ) used with SAPT0, SAPT2+, SAPT2+(3)/dmp2, and ALMO EDA2. Interestingly, while results for SAPT2+(3)/dmp2 and SAPT2+ showed more deviation from CCSD(T) with the smaller basis set (jun-cc-pVDZ), SAPT0+ actually showed less deviation than it did with the larger basis sets (aug-cc-pVTZ and aug-cc-pVDZ). SAPT0/jun-cc-pVDZ was previously reported to describe base-stacking interactions with sufficient accuracy [51].

The ALMO EDA2 results did not display basis set dependence. The SAPT2+(3)/dmp2 with aug-cc-pVDZ results were closest to ALMO EDA2, but SAPT2+(3)/dmp2 with aug-cc-pVTZ and SAPT2+ with aug-cc-pVDZ results were also reasonable. The interaction energy of the BenCl-BenCl complex was the most challenging to the various theoretical methods. Further analysis for this deviation is needed to tackle the inconsistent and inaccurate interaction energy for the halogen-substituted benzene and heterocycles in general, especially BenCl.

Based on these results, the interaction energies of the main nucleotide component fragments were then studied using ALMO EDA2 (B97M-V, original scheme), SAPT0 with jun-cc-pVDZ, SAPT2+ with aug-cc-pVDZ, and SAPT2+(3)/dmp2 with either aug-cc-pVTZ or aug-cc-pVDZ. The fragment interactions consisted of water in varying positions with 1. C2′-endo deoxyribose and C3′-endo deoxyribose sugars, 2. negatively charged dimethyl phosphate (DMP gt, DMP tt, and DMP gg), and 3. Nucleobases (Thymine, Adenine, Uracil, Cytosine, and Guanine). Additionally, nucleobase interactions via intrastrand base-stacking and interstrand hydrogen bonding were investigated.

Interaction energies and energetic components of several configurations of water interacting with the most common forms of the sugars present in nucleic acids, C2′-endo deoxyribose and C3′-endo ribose, were evaluated. The water was also translated to various distances from the sugar to obtain interaction energy profiles. The most stable configurations for water interacting with the sugar moieties (Deoxyribose-Wat1 and Wat5; Ribose-Wat1, Wat3, and Wat9) showed water forming two hydrogen bonds, including one with an oxygen of the 5-membered ring. SAPT2+(3)/dmp2 with aug-cc-pVDZ was the most inconsistent of the methods used to evaluate the sugar-water interactions despite its performance in the base-stacking energy evaluations. SAPT0 with jun-cc-pVDZ, SAPT2+(3)/dmp2 with aug-cc-pVTZ, and ALMO EDA2 second generation were consistent with each other for the interaction energies. The SAPT and ALMO energy decompositions were similar; although dispersion was more negative with SAPT than with ALMO, overall interaction energies remained similar due to the induction (polarization + charge transfer) term being more negative with ALMO. Exchange repulsion was the dominant positive component; electrostatics was the dominant attractive component. Induction was responsible for ~30–40% of the electrostatic energy component.

A similar process was conducted for water interacting with DMP. This interaction was found to be somewhat conformationally dependent, with DMP tt-Wat1 being the most stable. In this conformation, water forms two hydrogen bonds with the phosphate group, rather than one with the phosphate group and another with the attached –OCH_3_, as seen in DMP tt-Wa9 and Wat10. The interaction energies were again consistent across the methods, with the exception of SAPT0/jun-cc-pVDZ. Due to this poor performance in describing one of the representatives of the backbone, SAPT0/jun-cc-pVDZ is not recommended, despite its sufficient performance in describing the small molecule hydrogen bonding and base stacking and the sugar-water interaction. Energy decomposition again showed that electrostatics were the largest favorable contribution for most of the DMP-Wat configurations, partially counterbalanced by the unfavorable exchange-repulsion component.

Next, water-nucleobase interactions were investigated. Varying starting structures were chosen to ensure that waters could interact with each of the polarizable groups and then optimized to obtain equilibrium geometries. The waters were then translated along a vector connecting the water and nucleobase centers of mass, and interaction energies were calculated at various distances. The SAPT2+(3)/dmp2 with aug-cc-pVTZ and ALMO EDA2 showed good agreement with each other. The SAPT2+(3)/dmp2 with aug-cc-pVDZ results showed the largest deviation from the other methods at short distances; we chose not to continue with this method. Thymine and adenine were chosen as representatives of pyrimidines and purines, respectively, for the decomposition of the interaction energies with water. The results for both nucleobases with SAPT2+(3)/dmp2 and ALMO EDA2 showed exchange (SAPT) and repulsion (ALMO) as the dominant component, partially counterbalanced by the attractive electrostatic component.

Nucleobase interstrand (hydrogen bonding) and intrastrand (π-stacking) in B-DNA form were investigated using SAPT2+(3)/dmp2 and ALMO EDA2 with B97M-V. Dispersion was dominant in the intrastrand GG and AA base stacking interactions, partially counterbalanced by exchange/repulsion. Electrostatics were the third most influential component for both, although they were stabilizing for GG and destabilizing for AA. Induction was small but stabilizing for both. For both the interstrand G:C and A:T, hydrogen bonding interactions, dispersion, and electrostatics were the two largest components. However, for G:C, the attractive electrostatics component dominated, and dispersion had a smaller stabilizing contribution; for A:T, dispersion was dominant, and the electrostatic component was much smaller in magnitude. This may be due to the A:T base pair having two hydrogen bonds in comparison to three for the G:C base pair. Induction gave a small addition to stabilizing for both, although it was larger than the destabilizing repulsion for G:C but smaller than the repulsion for A:T. The two methods showed similar results overall, with the largest deviation in the description of dispersion for the intrastrand AA base stacking.

SAPT2+(3)/dmp2 and ALMO EDA2 were found to be the most consistent with each other with respect to the obtained interaction energies. Most of the difference observed between the two methods was in the stacked interaction energies of the nucleobases. Another important point to consider is the computational cost of the calculations; ALMO EDA2 is faster on average than SAPT2+(3)/dmp2 with similar accuracy.

## 4. Materials and Methods

### 4.1. Preparation of Structures

The canonical structures of the nucleotides were constructed using Nucleic.exe implemented in Tinker8 [59,60]. C2′-endo deoxyribose sugar was extracted from B-DNA geometry, while C3′-endo ribose sugar was extracted from A-RNA geometry. The purine and pyrimidine nucleobases were obtained from the B-DNA geometry of the nucleic acid as indicated by Tinker8 [59,60]. The three dominant conformations of DMP (DMP gg, DMP gt, and DMP tt) were constructed using Pymol 2.5.4 [17]. The structures (and corresponding nomenclature) of the B-DNA inter- and intrastrand nucleobases were those published by Sherill et al. [25].

After obtaining the canonical structures of the nucleotide components, water molecules were placed at various positions surrounding the structures following the aISS workflow [58]. The aISS workflow is based on a search for the lowest energy geometry for non-covalent interaction between two fragments. The first step in the workflow is a calculation of the electronic structure properties using semi-empirical GFNn-xTB [61,62] followed by a grid search based on xTB-IFF [63] interaction energies of the two fragments. In other words, several geometries are generated for the two interacting fragments by moving the first fragment around the second one. Then, the interaction energy is evaluated using xTB-IFF. The structures generated from the grid search are then subjected to genetic optimization followed by a final GFNn-xTB or GFN-FF optimization.

Fifteen different conformations of the water molecule interacting with the different constituents were generated (nucleobase-water, sugar moiety-water, and DMP-water). Three different conformations of the water molecule probe were selected based on the docking score and the uniqueness of the water molecule position. In other words, the position of the water molecule was chosen to cover the entire conformational space around the counter-interacting moieties and to interact with all the polarizable groups present in the structure.

The selected three complexes with the water molecule probe were subjected to energy minimization at the MP2/6-31G* theory level using GAUSSIAN16 [64]. This was followed by the translation of the water molecule probe along a vector connecting the center of mass of the water molecule and the center of mass of the interacting moiety. The various distances corresponded to multiples of the equilibrium distance of the optimized structures. The water molecule was translated to five or six positions corresponding to 0.90, 0.95, 1.0, 1.05, 1.1, and 1.3 times the equilibrium distance between the center of mass of the water molecule and the center of mass of the interacting counterpart. These distances do not necessarily reflect the interacting distances between the two molecules. This translation of the water probe helped us to generate an interaction energy curve of the nucleobase-water, sugar moiety-water, and DMP-water, which helped us identify the local minimum of each structure.

### 4.2. Ab Initio Calculations

The interaction energies and components of the nucleobase-water, sugar moiety-water, DMP-water, and inter-/intra-strand base pair complexes were computed using both the original and the new decomposition energy schemes of ALMO (Absolutely Localized Molecular Orbital) EDA2 [28,29,30], implemented in the QCHEM 6.1 package [54]. ALMO EDA decomposes the interaction energy into three main terms: frozen density, polarization, and charge transfer. The frozen density core includes electrostatics, pauli-repulsion, and dispersion. ALMO EDA2 resolved the shortcomings of ALMO EDA, in which the polarization showed basis set dependence, becoming contaminated by charge transfer when larger basis sets were used. Two energy decomposition schemes are popularly used with ALMO EDA2: the first is the original energy decomposition scheme, which includes the Frozen energy decomposition with ALMO polarization (AO-block-based); the new energy decomposition scheme involves Frozen energy decomposition with nDQ-FERF polarization. Both were tested on a set of small molecules to investigate performance in computing the interaction energies of the nucleotides.

Different levels of SAPT (Symmetry-Adapted Perturbation Theory): SAPT0, SAPT2+, and SAPT2+(3)/dmp2 [26,56,57,65,66] implemented in the Psi4 package [27] were also used to compute the interaction energies. SAPT determines the interaction energy between two fragments directly by decomposing the energy into four physically meaningful terms: electrostatics, exchange, induction, and dispersion. SAPT0 mainly depends on error cancellation. Higher-order SAPT depends on including several corrections; for example, SAPT2+ involves second-order correction for the monomeric fluctuations and dispersion energy correction by adding the effect of the intramolecular correlation [55]. SAPT2+(3)/dmp2 includes higher-order perturbative terms and variational correction terms.

## 5. Conclusions

QM benchmarks for calculations involving nucleotides are essential for developing forcefields that more accurately describe the non-covalent interactions preserving the complex structure of nucleic acids. Upon testing five different protocols, we recommend using SAPT2+(3)/dmp2 with aug-cc-pVTZ and ALMO EDA2 with B97M-V and aug-cc-pVTZ as high-quality ab initio methods for studying the non-covalent interactions of the nucleic acid. With SAPT, a higher theory level and/or larger basis set did not necessarily give more accuracy. It is hard to know which combination would be best for a given system. In contrast, ALMO EDA2 did not show dependence on theory level or basis set; additionally, it is faster.

The attractive induction and dispersion were found to be responsible for stabilizing the interactions of the neutral moieties (sugar moiety and nucleobase) with water due to the cancellation between the large Exchange-Pauli repulsion component and the attractive electrostatic components. Dispersion was found to be the dominant energetic component for the intrastrand base stacking interactions of both GG and AA, counterbalanced partially by exchange/repulsion. In the interstrand hydrogen-bonding interaction, the electrostatic component was dominant for G:C, but dispersion was dominant for A:T. Repulsion was the only destabilizing component, but it was fairly small. The energy decomposition methods showed similar results for all the interactions studied, lending additional credibility to the results. Furthermore, these results highlight the importance of an accurate description of electrostatics for molecular potentials describing nucleic acids.

In this work, we focused on intermolecular interactions of the fragments (ribose, phosphate, and bases). As we assemble the full polarizable force field (AMOEBA) models for complete nucleotides, additional QM calculations will be carried out on larger and more “realistic” model compounds (such as nucleoside or phosphate with ribose) in gas phase and implicit solvents in addition to investigation of the conformational energy surfaces with regard to dihedral angles and sugar puckering.

## Data Availability

The scripts used to generate the figures in the manuscript are uploaded as Supplementary Materials.

## Supplementary Materials

The following supporting information can be downloaded at https://www.mdpi.com/article/10.3390/molecules29143258/s1.
